# Plaque characteristics after endovascular treatment in patients with intracranial atherosclerotic disease

**DOI:** 10.1186/s41016-022-00302-3

**Published:** 2022-12-02

**Authors:** Shun Zhang, Junjie Wang, Jun Lu, Peng Qi, Shen Hu, Ximeng Yang, Kunpeng Chen, Daming Wang

**Affiliations:** 1 Department of Neurosurgery, Beijing Hospital, National Center of Gerontology, Dongcheng, Beijing, China; 2 Graduate School of Peking Union Medical College, Dongcheng, Beijing, China

**Keywords:** Intracranial atherosclerotic disease, Endovascular treatment, Plaque characteristics, Follow-up, HRMRI

## Abstract

**Background:**

Endovascular treatment (EVT) is an alternative option for symptomatic intracranial atherosclerotic disease (ICAD). However, the effect of EVT treatment on ICAD plaques is still unclear. This study describes the ICAD plaque characteristics after EVT treatment and analyzes the effect of different EVT treatments on plaque characteristics.

**Method:**

From 2017 January to 2022 January, ICAD patients who underwent endovascular treatment and had follow-up high-resolution magnetic resonance image (HRMRI) were enrolled in the study. Multiple plaque characteristics, including plaque enhancement, plaque burden, were measured based on preoperative, and follow-up HRMRI. Plaque characteristics and postoperative plaque changes were analyzed between different treatment groups.

**Result:**

Finally, 50 intracranial atherosclerotic plaques in 45 patients were included. Including 28 male patients and 17 female, media age 63.0 years old. Among 50 plaques, 41 received percutaneous angioplasty (including 22 plain balloons and 19 drug-coated balloons (DCB)) and the other 9 underwent stenting. Stenosis rate, plaque burden and eccentricity index at the lesion site were significantly decreased after EVT compared with preoperative periods (*p* <0.001). And only the DCB group showed a significant reduction in plaque enhancement at follow-up (*p* < 0.001). No significant preoperative and postoperative changes in other plaque characteristics were found.

**Conclusion:**

EVT treatment could compromise the characteristics of intracranial periarterial atherosclerotic plaques, and DCB treatment may result in a reduction in plaque enhancement after treatment.

## Background

Intracranial atherosclerotic disease (ICAD) is one of the major cause of stroke and is associated with 30–50% of ischemic strokes in Asian populations [1]. Intracranial atherosclerotic plaques are at the key of ICAD lesions and contribute to the development of ischemic strokes through a variety of mechanisms, including: progression of atherosclerotic plaque leading to increased luminal stenosis followed by distal hemodynamic disturbance; plaque rupture leading to increased luminal stenosis or arterial-arterial embolism; a atherosclerotic plaque affecting the opening of small penetrating arteries leading to their occlusion [2]. Therefore, an advanced understanding of intracranial atherosclerotic plaques is important for the treatment of ICAD lesions as well as the prevention and treatment of strokes [3, 4].

Endovascular treatment (EVT) including percutaneous angioplasty (PTA), drug-coated balloon (DCB), and stenting have become common options for the treatment of ICAD; especially for patients who are not well treated with medical therapy, it can immediately decrease luminal stenosis and effectively improve perfusion. However, one of the main problems of PTA and stenting is the occurrence of postoperative restenosis, and studies have shown that the restenosis rate can be as high as 27.6% in the first year after stenting [5], long-term follow-up after balloon dilation shows a restenosis rate of approximately 30% [6, 7]. Studies suggest that mechanical stress during treatment injured the vessel wall, and the secondary inflammatory response promotes smooth muscle cell proliferation and vascular endothelial proliferation, which are the main causes of restenosis [8].

High-resolution MRI (HRMRI) widely used in the diagnosis of many various intracranial vascular lesions [9], which can effectively identify intracranial atherosclerotic plaques with good interreader agreement [10]. HRMRI can provide features of atherosclerotic plaques such as eccentricity, plaque burden, and degree of plaque enhancement [11–14]. Several studies have preliminarily confirmed that plaque characteristic in HRMRI can reflect the histopathological features of intracranial atherosclerotic plaques [15, 16], And these plaque features are believed to be closely associated with the occurrence of ischemic events or impaired distal blood flow in ICAD patients.

However, few studies have been reported about the application of HRMRI to observe plaque characteristics in patients after EVT. In this study, we aimed to characterize the plaque in post-EVT disease and to analyze the effect of different treatments on the plaque.

## Methods

### Patient selection

Patients over 18 years old with symptomatic ICAD who underwent endovascular treatment and future HRMRI follow-up in our center between 2017 January and 2022 January were include in our study. The exclusion criteria were (1) patients with less than 70% stenosis. (2) Patients were proven to have non-atherosclerotic stenosis such as dissection and Moyamoya disease. (3) Patients with hyperacute stroke. (4) Patients who refused to participate in the experiment.

### Data collection and follow-up outcomes

Patients’ demographic information, perioperative data were obtained from the case system. Follow-up and preoperative HRMRI data of the patients were obtained from the imaging workstation. The degree of restenosis at follow-up was judged according to follow-up HRMR images, and the criteria was over 50% stenosis within or immediately adjacent (within 5 mm) to the treated segment and > 20% absolute luminal loss.

### Surgery and medical treatment

The endovascular treatment and perioperative management follow the same protocol as our pervious study.

In brief, digital subtraction angiography was performed for all the patients and the strategy of endovascular treatment was decided according to the site and characteristics of the target lesions and based on the experience and preference of the operators. Prior to the intervention, all the patients were under dual antiplatelet therapy (DATP) with aspirin and clopidogrel, dual antiplatelet therapy was maintained for at least 6 months, and aspirin or clopidogrel alone was continued daily afterward. Long-term management of individual medical risk factors, such as blood pressure, cholesterol, and diabetes mellitus, was implemented [17].

### Imaging protocol

High-resolution magnetic resonance was performed with a 3.0-T MR scanner (Achieva; Philips Health care, Best, The Netherlands) with a 16-channel NV coil and the same parameters as our previous research was used.

The HR-MRI sequences included 3D time of flight MRA and pre and post-contrast T1W imaging (VISTA). The parameters were as follows for time-of-flight MRA: repetition time = 25 ms; echo time = 3.45 ms; field-of-view = 180 mm × 180 mm; and acquired resolution = 0.55 mm × 0.55 mm × 1.1 mm. For T1W imaging, the parameters were repetition time = 800 ms; echo time = 18 ms; field of view = 200 mm × 180 mm × 40 mm; and acquired resolution = 0.6 mm × 0.6 mm × 0.6 mm. Gadoteric acid meglumine (Dotarem; Guerbet, Aulnay-sous-Bois, France) was intravenously injected (0.1 mmol/kg of bodyweight). T1W imaging was repeated 5 min after injection [17].

### Imaging analysis

An experienced neuroradiologist evaluated the quality of all HRMR images. Images with poor quality that cannot complete subsequent measurements were excluded from further analyses.

Plaque characteristics were measured by two neuroradiologists on PACS workstation (Neusoft, Shenyang, China; version 5.5.0).

**Fig. 1:**
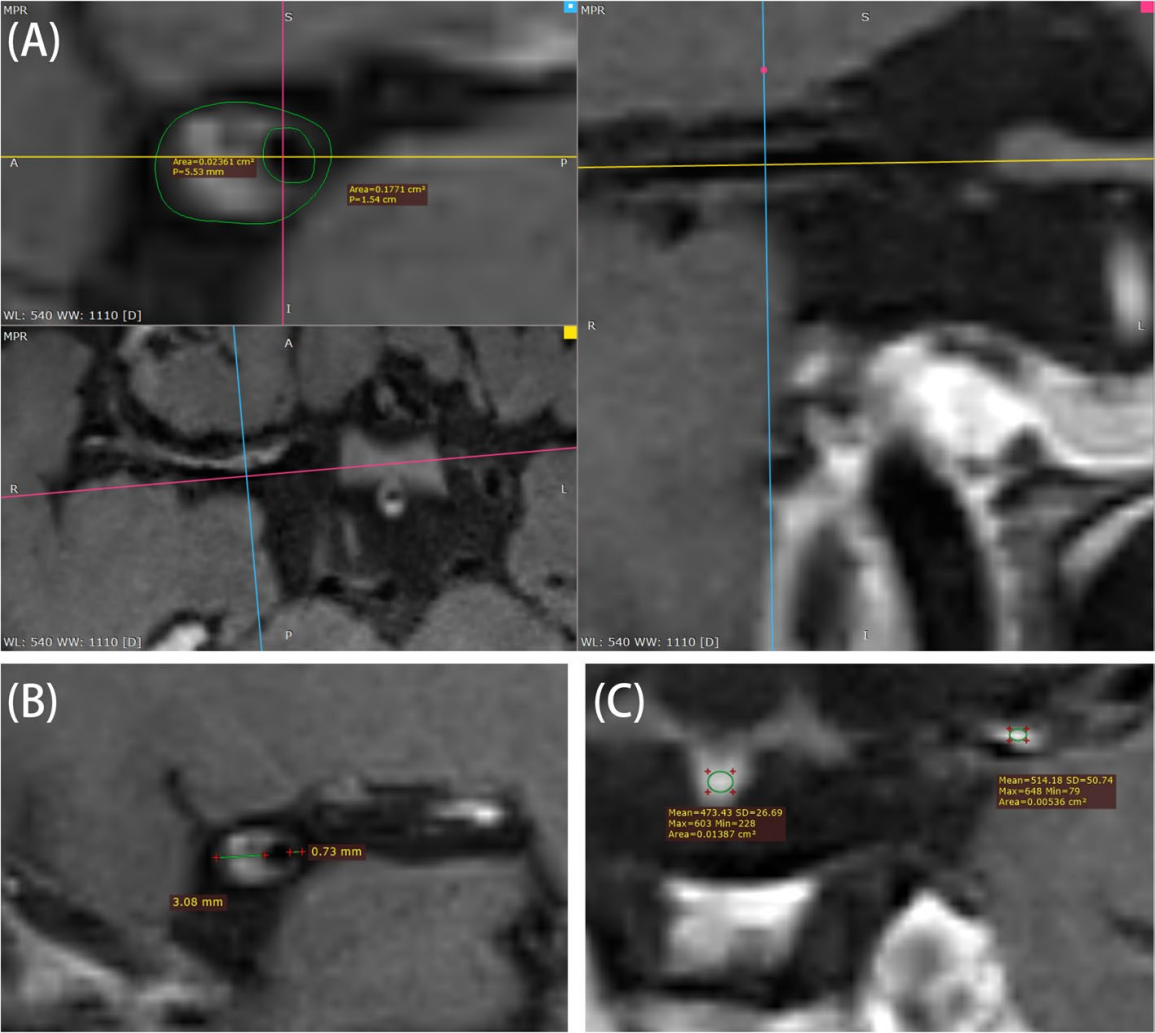
Illustration of measurement of outer wall area, inner wall area (**A**), eccentricity index (**B**), and plaque enchantment (**C**)

Quantitative MRI measurements included the following:

1. Outer wall area (OWA): outer wall area of blood vessels.
2. Lumen area (LA): inner wall area of blood vessels.
3. Wall area (WA): WA = OWA-LA.
4. Stenosis degree: stenosis degree = (1-LA stenosis/LA reference). The reference site was defined as the normal vessel segment proximal or distal to the maximal stenotic site.
5. Plaque burden: plaque burden = (1-lumen area/vessel area) × 100% [18].
6. Remodeling ratio (RR): RR = OWA stenosis/LA reference. RR > 1.05 was considered as positive remodeling, 0.95 ≤ RR ≤ 1.05 as intermediate, and RR < 0.95 as negative [19].
7. Plaque enhancement: plaque enhancement was divided into three levels: grade 0: ≤ the degree of enhancement of the normal intracranial vascular wall; grade 1: between the degree of enhancement of the normal vessel wall and the degree of enhancement of the pituitary infundibulum; grade 2: ≥ the degree of enhancement of the pituitary infundibulum [20].
8. Eccentricity index (EI): EI = 1-minimum wall thickness/maximum wall thickness. A lesion was defined as eccentric if the index was ≥ 0.5 and as concentric if < 0.5 [21] (Fig. 1).

### Statistical analysis

The SPSS software (version 26.0; SPSS Inc., Chicago, IL, USA) was used for data analyses. Mean ± standard deviation (SD) was used to present continuous variables; the frequency and percentage were used for categorical variables. The continuous variables according to normal distribution were compared before and after operation using repeated measures *t* test and between groups using one-way ANOVA; continuous variables not according to normal distribution were compared before and after operation using Wilcoxon conformity rank test and between groups using Kruskal-Wallis *H* test; categorical variables were compared using chi-square test; Kendall’s tau-b correlation coefficient was used to compare correlations between two variables; all statistical tests were two-sided, with *P* < 0.05 defined as a statistical difference.

## Results

### Patient characteristics

Finally, 50 intracranial atherosclerotic plaques in 45 patients were included. Including 28 male patients and 17 female, media age 63.0 years old. Among 50 plaques, 41 received percutaneous angioplast, including 22 with plain balloons and 19 with drug-coated balloons (DCB), and the other 9 underwent stenting. Most treated lesions were located in the anterior circulation, as shown in Table 1.

**Table 1:** Characteristics of patients underwent endovascular recanalization and received HR-MR follow-up

Characteristics	All (***n*** = 50)	PTA (***n*** = 22)	DCB (***n*** = 19)	STENTING (***n*** = 9)	Coefficient	***P***
Gender (male)	**28**	**13**	**11**	**4**		***0.747***
Age (years)	**63.0 (57.0–69.0)**	**61.0 (55.3–65.0)**	**64.0 (62.0–74.0)**	**67.5 (65.3–70)**		***0.127***
**Location**						
**MCA**	**24 (48.0%)**	**10 (45.5%)**	**11 (57.9%)**	**3 (33.3%)**		**0.534**
**ICA**	**10 (20.0%)**	**3 (13.6%)**	**5 (26.3%)**	**2 (22.2%)**		
**BA**	**12 (24.0%)**	**7 (31.8%)**	**2 (10.5%)**	**3 (33.3%)**		
**VA**	**4 (8.0%)**	**2 (9.1%)**	**1 (5.3%)**	**1 (11.1%)**		
**Side**						
**Left**	**20 (52.6%)**	**8 (53.3%)**	**10 (58.8%)**	**2 (66.7%)**		**0.581**
**Right**	**18 (47.4%)**	**7 (46.7%)**	**7 (41.2%)**	**4 (33.3%)**		
**Follow-up time (days)**	**319.68 ± 226.81**	**252.36 ± 200.85**	**311.55 ± 185.60**	**501.40 ± 286.20**		**0.018**
**Stenosis degree (%)**	**48.92 ± 27.32**	**61.33 ± 24.08**	41.92 ± 28.91*	33.39 ± 18.92*	***F*** **= 5.042**	**0.010**
**Plaque burden**	**0.74 ± 0.19**	**0.80 ± 0.10**	0.69 ± 0.22*	0.59 ± 0.13*	***K*** **= 14.234**	**0.001**
**RR**	**0.89 ± 0.31**	**0.87 ± 0.28**	**0.89 ± 0.28**	**0.97 ± 0.44**	***K*** **= 0.056**	**0.973**
**EI**	**0.49 ± 0.25**	**0.49 ± 0.26**	**0.48 ± 0.24**	**0.49 ± 0.27**	***K*** **= 0.052**	**0.974**
**Plaque enhancement**						
**Grade 0**	**13 (26.0%)**	**2 (9.1%)**	**10 (52.6%)**	**1 (11.1%)**	***K*** **= 14.541**	**0.001**
**Grade 1**	**22 (44.0%)**	**9 (40.9%)**	**8 (42.1%)**	**5 (55.6%)**		
**Grade 2**	**15 (30.0%)**	**11 (50.0%)**	**1 (5.3%)**	**3 (33.3%)**		
**Eccentric**						
**No**	**23 (46.0%)**	**11 (50.0%)**	**7 (36.8%)**	**5 (55.6%)**	*χ*^2^ **= 1.114**	**0.573**
**Yes**	**27 (54.0%)**	**11 (50.0%)**	**12 (63.2%)**	**4 (44.4%)**		
**Remodeling pattern**						
**Negative**	**34 (68.0%)**	**15 (68.2%)**	**12 (63.2%)**	**7 (77.8%)**	*χ*^2^ **= 1.760**	**0.780**
**Intermediate**	**5 (10.0%)**	**2 (9.1%)**	**3 (15.8%)**	**0 (0.0%)**		
**Positive**	**11 (22.0%)**	**5 (22.7%)**	**4 (21.0%)**	**2 (22.2%)**		
**Restenosis**						
**No**	**36 (72.0%)**	**14 (63.6%)**	**16 (84.2%)**	**6 (66.7%)**	*χ*^2^ **= 2.296**	**0.317**
**Yes**	**14 (28.0%)**	**8 (36.4%)**	**3 (15.8%)**	**3 (33.3%)**		

*Compare with DCB group, *p* < 0.05

The median radiological follow-up was 319.9 ± 226.81 days. On follow-up, the overall restenosis rate was 28.0% (14/50). Of these 14 patients with restenosis, 2 patients were in the stenting group (30.0%, 3/9). The other 12 cases were in the angioplasty group (26.8%, 11/41). One patient in the stenting group suffered from symptomatic restenosis, and he received further revascularization with the insertion of another stent in the intracranial vertebral artery. The other 13 patients were asymptomatic and received medical treatment. The DCB subgroup showed a lower rate of restenosis than the percutaneous transluminal angioplasty (PTA) subgroup [15.8% (3/19) vs. 36.4% (8/22)] (Table 1).

### Plaque characteristic after endovascular treatment

In this study, plaque characteristics were measured in 50 lesions on follow-up HRMRI images after EVT. Follow-up HRMRI showed an overall mean stenosis rate of 48.92 ± 27.32% at the postoperative lesion. The mean stenosis rates were 41.92 ± 28.91% and 33.39 ± 18.92% in the DCB and stenting groups, respectively, which were lower than the 61.33 ± 24.08% in the PTA group (*P* = 0.01)。The plaque burden were 0.69 ± 0.22, 0.59 ± 0.13, and 0.84 ± 0.10 in the DCB, stenting, and PTA groups, respectively, and the postoperative plaque burden were lower in the DCB and stent implantation groups than in the PTA group (*P* < 0.001). Among the 50 lesions, 26.0% (13/50) of the plaques had grade 2 enhancement, 44% (22/50) had grade 1 enhancement, and 26% (13/50) had grade 0 enhancement. In the DCB group, the proportion of grade 0 enhanced plaques was 52.6% (10/19), which was higher than that in the stenting group (11.1%, 1/9) and the PTA group (9.1%, 2/22), while the proportion of grade 2 enhanced plaques in the PTA group was 50.0%(11/22), which was higher than that in the stenting group (33.3%, 3/9) and the DCB group (5.3%, 1/19). There was no statistical difference between the PTA, DCB, and stenting groups in terms of whether the lesion was an eccentric stenosis, EI, RR, and vascular remodeling pattern after EVT (Table 1). Univariate analysis was preformed to explore the relationship between postoperative plaque characteristics and restenosis, but no correlation was found.

### Changes in plaque characteristics pre and postoperative

Twenty-eight plaques with both preoperative and follow-up HRMRI images, the preoperative plaque characteristics, were summarized in Table 2. When plaque characteristics were compared between pre- and postoperative periods, the results showed that the stenosis rate, plaque burden and EI at the lesion site were significantly decreased after EVT compared with preoperative periods (*p* < 0.001), as well as the degree of plaque enhancement was also decreased (*p* = 0.009) (Table 3).

**Table 2:** Summary of preoperative plaque characteristics

	All(***n*** = 34)	PTA(***n*** = 14)	DCB(***n*** = 15)	Stenting(***n*** = 5)
**Stenosis degree (%)**	**68.68 ± 17.97**	**74.78 ± 20.53**	**67.15 ± 14.24**	**66.21 ± 15.89**
**Plaque burden**	**0.89 ± 0.06**	**0.91 ± 0.07**	**0.89 ± 0.04**	**0.84 ± 0.06**
**RR**	**0.91 ± 0.39**	**0.99 ± 0.38**	**0.74 ± 0.23**	**1.23 ± 0.59**
**EI**	**2.13 ± 0.91**	**1.78 ± 0.77**	**2.11 ± 0.69**	**3.14 ± 1.22**
**Enhancement**				
**Grade 0**	**0 (0.0%)**	**0 (0.0%)**	**0 (0.0%)**	**0 (0.0%)**
**Grade 1**	**21 (61.8%)**	**9 (64.3%)**	**7 (46.7%)**	**5 (100.0%)**
**Grade 2**	**13 (38.2%)**	**5 (35.7%)**	**8 (53.3%)**	**0 (0.0%)**
**Eccentric**				
**No**	**13 (38.2%)**	**7 (50.0%)**	**5 (33.3%)**	**1 (20.0%)**
**Yes**	**21 (61.8%)**	**7 (50.0%)**	**10 (66.7%)**	**4 (80.0%)**
**Remodeling pattern**				
**Negative**	**21 (61.8%)**	**8 (57.1%)**	**12 (80.0%)**	**1 (20.0%)**
**Intermediate**	**5 (14.7%)**	**1 (7.2%)**	**2 (13.3%)**	**2 (40.0%)**
**Positive**	**8 (23.5%)**	**5 (35.7%)**	**1 (6.7%)**	**2 (40.0%)**

**Table 3:** Changes in plaque characteristics pre and postoperative

Characteristics		Preoperative(***n*** = 34)	Postoperative(***n*** = 34)	***p***
**Stenosis degree (%)**		**68.68 ± 17.97**	**47.43 ± 28.04**	**< 0.001**
**Plaque burden**		**0.89 ± 0.06**	**0.75 ± 0.19**	**< 0.001**
**RR**		**0.91 ± 0.39**	**0.95 ± 0.32**	**0.584**
**EI**		**2.13 ± 0.91**	**0.46 ± 0.26**	**< 0.001**
**Plaque enhancement**				
**Grade 0**		**0 (0.0%)**	**11 (32.4%)**	**0.009**
**Grade 1**		**21 (61.8%)**	**14 (41.1%)**	
**Grade 2**		**13 (38.2%)**	**9 (26.5%)**	
**Eccentric**				
**No**		**13 (38.2%)**	**19 (55.9%)**	**0.145**
**Yes**		**21 (61.8%)**	**15 (44.1%)**	
**Remodeling pattern**				
**Negative**		**21 (61.8%)**	**20 (58.8%)**	**0.959**

### Influence of different treatment methods on plaque characteristics

**Fig. 2:**
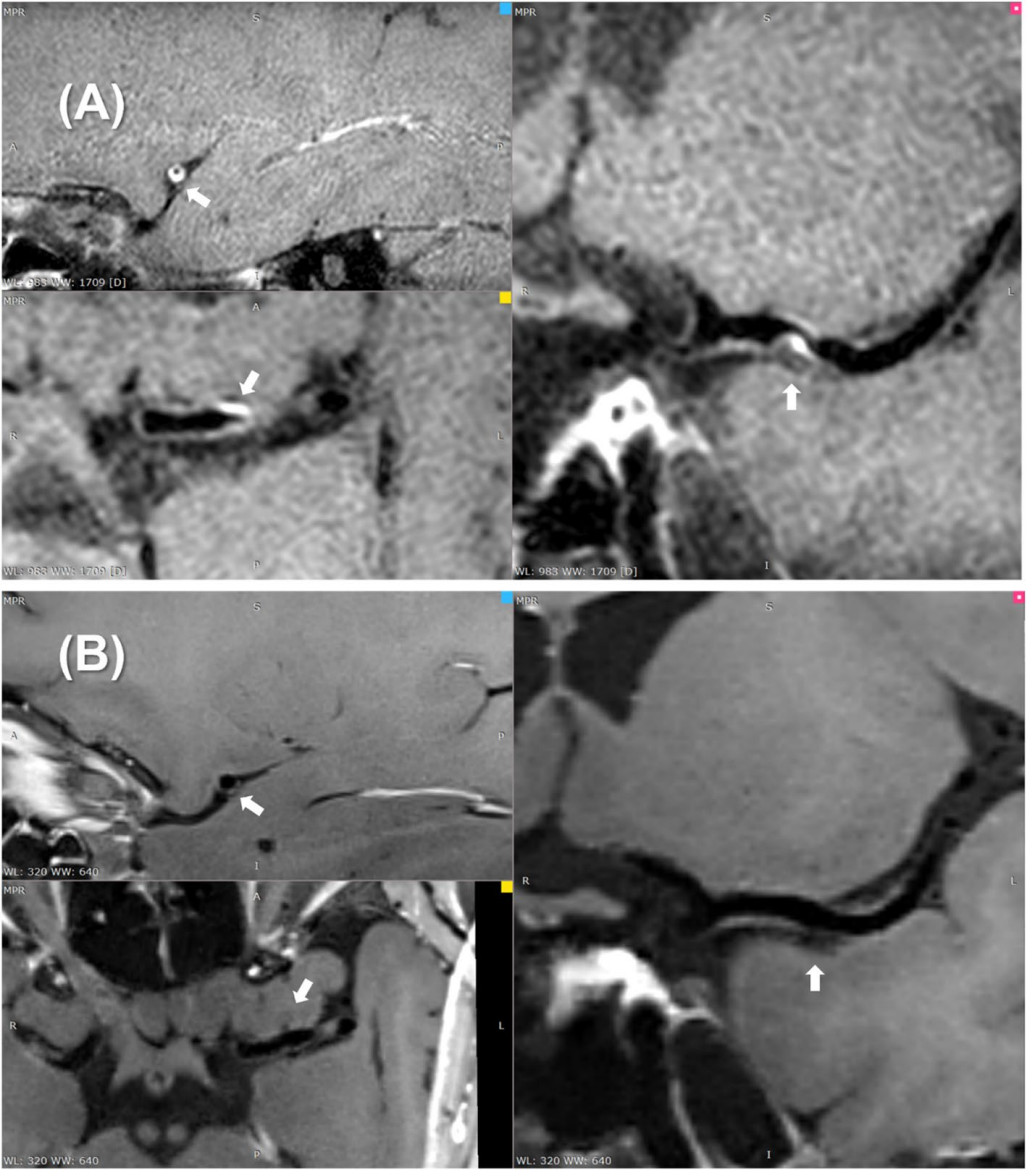
Preoperative (**A**) and 2-year follow-up HRMRI (**B**) of a patient with symptomatic ICAD treated by DCB. Significant reduction in plaque enhancement and plaque burden can be seen on the images

Repeated measures *t* tests, non-parametric analyses, and chi-square tests were used to analyze changes in plaque characteristics across treatment groups. The results revealed a significant decrease in stenosis degree and EI at postoperative follow-up for all three treatments compared to the preoperative period. The plaque burden at follow-up was decreased in the DCB and stent groups. The PTA group also showed a decreasing trend in plaque burden at follow-up, but there was no statistical difference. Only the DCB group showed a significant reduction in plaque enhancement at follow-up (*p* < 0.001) (Fig. 2), with the proportion of grade 2 enhanced plaques decreasing from 53.3 to 6.7% and the proportion of grade 0 enhanced plaques increasing from 0.0 to 60%. In contrast, no significant preoperative and postoperative changes in other plaque characteristics were found (Table 4).

**Table 4:** Changes in preoperative and postoperative plaque characteristics in each group

Characteristics	PTA (***n*** = 14)		***P***	DCB (***n*** = 15)		***P***	Stenting (***n*** = 5)		***P***
	Preoperative	Postoperative		Preoperative	Preoperative		Preoperative	Preoperative	
**Stenosis degree (%)**	**74.78 ± 20.53**	**58.99 ± 23.09**	**0.029**	**67.15 ± 14.24**	**42.27 ± 30.55**	**0.014**	**66.21 ± 15.89**	**24.91 ± 10.42**	**0.015**
**Plaque burden**	**0.91 ± 0.07**	**0.80 ± 0.10**	**0.121**	**0.89 ± 0.04**	**0.71 ± 0.22**	**0.008**	**0.84 ± 0.06**	**0.57 ± 0.11**	**0.011**
**RR**	**0.99 ± 0.38**	**0.94 ± 0.24**	**0.684**	**0.74 ± 0.23**	**0.90 ± 0.29**	**0.049**	**1.23 ± 0.59**	**1.13 ± 0.54**	**0.429**
**EI**	**1.78 ± 0.77**	**0.43 ± 0.28**	**0.001**	**2.11 ± 0.69**	**0.45 ± 0.26**	**0.000**	**3.14 ± 1.22**	**0.54 ± 0.26**	**0.010**
**Plaque enhancement**									
**Grade 0**	**0 (0.0%)**	**2 (14.2%)**	**0.705**	**0 (0.0%)**	**9 (60.0%)**	**0.001**	**0 (0.0%)**	**0 (0.0%)**	**0.157**
**Grade 1**	**9 (64.3%)**	**6 (42.9%)**		**7 (46.7%)**	**5 (33.3%)**		**5 (100.0%)**	**3 (60.0%)**	
**Grade 2**	**5 (35.7%)**	**6 (42.9%)**		**8 (53.3%)**	**1 (6.7%)**		**0 (0.0%)**	**2 (40.0%)**	
**Eccentric**									
**No**	**7 (50.0%)**	**9 (64.3%)**	**0.445**	**5 (33.3%)**	**7 (46.7%)**	**0.456**	**1 (20.0%)**	**3 (60.0%)**	**0.197**
**Yes**	**7 (50.0%)**	**5 (35.7%)**		**10 (66.7%)**	**8 (53.3%)**		**4 (80.0%)**	**2 (40.0%)**	
**Remodeling pattern**									
**Negative**	**8 (57.1%)**	**8 (57.1%)**	**0.801**	**12 (80.0%)**	**9 (60.0%)**	**0.443**	**1 (20.0%)**	**3 (60.0%)**	**0.223**
**Intermediate**	**1 (7.2%)**	**2 (14.3%)**		**2 (13.3%)**	**3 (20.0%)**		**2 (40.0%)**	**0 (0.0%)**	
**Positive**	**5 (35.7%)**	**4 (28.6%)**		**1 (6.7%)**	**3 (20.0%)**		**2 (40.0%)**	**2 (40.0%)**	

## Discussion

To our knowledge, this is the first study of plaque characteristics on follow-up of ICAD treated by endovascular treatment. HRMRI can provide a reliable picture of plaque characteristics after EVT, and EVT leads to changes in plaque burden and plaque enhancement. In addition, there was heterogeneity in plaque characteristics and plaque changes between treatment groups, indicated that the effect of different treatments on plaque can be different.

Intracranial arterial plaque enhancement have been proved related to plaque inflammatory response and neovascularization and regarded as an imaging marker of criminal plaque. In our study, DCB group showed a significant decrease in plaque enhancement at follow-up compared to preoperative (*p* < 0.001). That could be related to local inflammatory responses caused by vascular injury after transluminal angioplasty stenting and percutaneous transluminal angioplasty. And paclitaxel used in DCB could inhibits that inflammatory response. This is consistent with the findings in animal models that DCB could decrease the atherosclerotic plaque inflammatory response [22]. Studies have shown that the degree of plaque enhancement in ICAD patients is strongly associated with the occurrence and recurrence of stroke [21, 23]. However, univariate analysis of our study showed no significant relationship between postoperative plaque characteristics and restenosis, which may be related to the small sample size. Studies with larger sample sizes are needed to explore the relationship between postoperative plaque enhancement and postoperative restenosis.

Plaque burden and eccentricity are also plaque characteristics that are strongly associated with stroke occurrence. Our study showed that plaque burden and eccentricity index at follow-up were significantly lower after EVT compared to preoperative (*p* < 0.001). Further subgroup analysis showed that plaque burden at follow-up was lower in the DCB and stenting groups compared to preoperative, with a greater decrease in plaque burden in the stent implantation group than in the DCB group. A decreasing trend in plaque burden is also shown in the PTA group, but there was no statistical difference. The study by RAN et al showed high plaque burden in the middle cerebral artery is an independent factor of recurrent ischemic stroke [24]. In Addition, some peripheral or coronary studies have shown that plaque burden decreases over the follow-up period after the application of DCB [25, 26]. Therefore, the change of plaque burden in different periods after EVT remains to be further observed.

In our study, no effect of the procedure on the rate of vascular remodeling and the pattern of vascular remodeling was observe, This may be related to the small sample size and the short follow-up period. Several extracranial artery-based studies have shown that lesions with a positive remodeling pattern have a higher probability of restenosis after stenting [26, 27]. Ma et al. showed that lesions with negative remodeling pattern after basilar artery stenting associated with penetrating stroke [28]. The relationship between vascular remodeling and EVT, as well as its relation to postoperative restenosis remains unclear and requires further study.

The present study has some limitations. First, this is a retrospective study with a few cases. That could lead to selection bias and less robust statistical results. Second, all plaque characteristics were measured manually. An automated plaque segmentation and measurement tool can improve measurement accuracy Third, ICAD is a disease that continuously develops. Our cross-sectional study could not fully reveal the development of plaques during follow-up. Further prospective large sample research was needed.

## Conclusion

In this study, we describe the plaque characteristics at the lesion site after EVT. The effect of different treatments on plaque characteristics was also analyzed. The results showed that patients had lower plaque burden after stenting and that DCB treatment resulted in a more pronounced reduction of atherosclerotic plaque enhancement, which may be related to the effect of paclitaxel.

## Data Availability

The data used to support the findings of this study are included within the article.
